# A Comprehensive Review on the Role of Interleukin-40 as a Biomarker for Diagnosing Inflammatory Diseases

**DOI:** 10.1155/2024/3968767

**Published:** 2024-03-01

**Authors:** Feryal Dabbagh-Gorjani

**Affiliations:** Department of Immunology, School of Medicine, Shiraz University of Medical Sciences, Shiraz, Iran

## Abstract

Interleukins are a group of proteins that have a wide range of complex functions and are believed to be involved in several diseases and conditions. In particular, interleukin-40 (IL-40) is a recently identified cytokine associated with B cells that was first introduced by Catalan et al. in 2017. This cytokine has several roles in the body, including functioning in the formation of B cells in the bone marrow, IgA production, and expression in the intestinal microbiome. Moreover, IL-40 appears to be involved in numerous autoimmune and inflammatory conditions, such as rheumatoid arthritis, systemic lupus erythematosus, primary Sjogren's syndrome, ankylosing spondylitis, type 2 diabetes, Graves' disease, and hepatic cell carcinoma. Our understanding of this molecule is quite restricted due to its novelty. However, because of its inflammatory characteristics, there is a high probability that it contributes to a variety of inflammatory disease complications. The aim of the present review is to highlight all available data on the importance of assessing IL-40 levels in human diseases up to now, which could be used as a diagnostic biomarker for the onset or progression of numerous inflammatory diseases.

## 1. Introduction

Interleukins are one of the most important proteins secreted by different cells in the body. They can participate in different crucial functions, such as migration, adhesion, maturation, and proliferation, and they help with the activation and differentiation of immune cells too [1–3]. Cytokines, like several other molecules, cause their effects by attaching to their specific receptors located on the membranes of their targets. Therefore, pairing the cytokine receptor with its ligand is the primary step to starting their activities, and thanks to the elucidation of this pathway, we now know more about how cytokines affect the emergence of immune responses [4].

According to the literature, at the end of the 20^th^ century, a group of cytokines had 30 members, and up until now, around 40 interleukins have been identified, and this number is constantly increasing [5]. Nowadays, with the rising use of gene arrays and other gene expression techniques, our knowledge of the universe of interleukins has also grown. As mentioned before, cytokines have a wide range of complex functions, and this causes them to affect other cells in a variety of ways, which can be the reason for overdepression in some cells and the emergence of disease. Autoimmune diseases, diabetes, neoplastic, neurological disorders, and kidney problems are examples of such conditions [6, 7]. Therefore, evaluating the levels of interleukins can be utilised as a great diagnostic marker for follow-up or diagnosis of numerous diseases [8, 9]. This study examines all available data on diseases in which the presence of interleukin-40 has been demonstrated.

## 2. Interleukin-40

**Figure 1:**
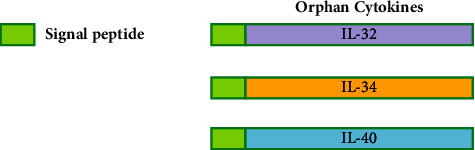
Graphical representation of orphan family cytokines created by Dabbagh-Gorjani. IL, interleukin.

In 2017, Catalan et al. introduced interleukin-40 (IL-40), a new cytokine associated with B cells that only mammals can express [10, 11]. They established that the C17orf99 gene is responsible for producing this small protein, and like IL-32 and IL-34, it belongs to the “orphan cytokine” group (Figure 1). An orphan cytokine is a small group of interleukins with distinct structural characteristics that do not have any homology with others [12]. It has been shown that TGF-*β* is a crucial factor for increasing the expression of IL-40 in activated B cells [13], but it seems that other factors could also enhance its expression that have not been discovered yet.

The first indication of its role in different inflammatory and autoimmune processes has been provided when the gene encoding the protein C17orf99 was identified as an autoimmune autoantigen associated with hepatitis B virus although it is not present in healthy controls [14].

Recently, an additional study has shown that the expression of C17orf99 downregulated in a human cell model of pneumonia after treatment with anti-inflammatory drugs, suggesting the presence of inflammation-related cytokines [15]. These findings confirm the possibility that IL-40 may be associated with inflammation and autoimmune disorders.

## 3. IL-40 Sources

Although fetal liver, bone marrow, and activated B cells are introduced as the main sources [11], IL-40 is currently thought to be produced by immune cells other than B cells. In one study about rituximab, an antibody against CD20, which is a marker of human B cells and is used to treat RA patients by reducing their B cells, scientists showed that it also results in a decrease in IL-40 [16]. But in the next stage of the experiment, when serum samples from RA patients were analyzed, they discovered that only 70% of IL-40 was lowered by this treatment [16]. Therefore, these data suggested that other immune cells that are associated with RA, such as neutrophils, T cells, and macrophages, caused the remaining 30% of IL-40 production [16]. Supporting this result, researches showed that neutrophils from arthritis patients are one of the important sources of interleukin-40 production during the early stages of the disease [17]. However, another study revealed that CD4+, CD8+ T cells, and CD68+ macrophages are additional producers of IL-40 in pSS patients, supporting the hypothesis that other immune cells may also create IL-40 [18]. Therefore, it is not just secreted by B cells and for the development of appropriate treatments to target this inflammatory cytokine in the future, it is crucial to identify the main source from which this cytokine is secreted in various diseases.

## 4. Physiologic Functions of IL-40

Although IL-40 is a new cytokine and we need to acquire more information about it, it is considered that it plays different roles in the body. The first function that IL-40 became well known for was its key role in the formation of B cells in bone marrow (BM) [10]. Studies have revealed that IL-40 knockout mice develop abnormal B cell populations [10]. In addition, it has been demonstrated that except for BM, the mammary gland secretes IL-40, which controls IgG secretion locally to maintain normal B cell function [5, 12].

Another interesting discovery in one study was the correlation between IL-40 and the composition of the intestinal microbiome in mice [10]. To put it another way, IL-40, via its effects on B cell activity, increases IgA production and can affect the population of bacteria in the mucus of the intestine [10].

In compliance with this experiment, researchers showed that IL40-/-mice have an incredibly low IgA concentration in their milk, and this was not only in the mammary gland, and they also confirmed a general IgA deficiency in IL40-/-mice. The IgA deficiency in this experiment showed that IL-40 has a great role in the normal function of B cells in the periphery [12]. Overall, although scientists have been unable to illustrate the function of IL-40 in class switching, these data demonstrate that the presence of IL-40 during the Ig-A responses is vital.

## 5. The Role of IL-40 in Disease

In recent years, scientists have shown the importance of B cells' activities and their cytokines in several studies. It is argued that IL-40 could be considered as an important diagnostic biomarker in B cell-mediated diseases. This hypothesis was strengthened when researchers discovered that human B cell lymphoma cell lines (OCI-Ly1) constitutively express IL-40, pointing to a potential function for IL-40 in illnesses connected to human B cells [12, 19].  Table 1 shows a summarized characteristic of IL-40 and in the following, we will examine all available information on the role of this cytokine in various diseases.

### 5.1. IL-40 and Lymphoma

Activation of human peripheral blood cells with anti-CD40 Ab plus IL-4 in human illustrated an upregulation in the gene expression of C17orf99/IL-40 [10]. On the other hand, studies on human non-Hodgkin B cell lymphoma lines, OCI-Ly1, HL-2, and Val, showed that they persistently produce IL-40 and this cytokine may play an important role in pathogenesis of human lymphoma [10]. To comply with this, the expression of TSPAN33 Ag, B cell activation marker, on IL-40^+^ lymphomas were observed [20] which confirmed the fact that lymphomas are activated forms of B cells producing IL-40 [10]. However, Ovidiu Farc in 2019 could not show the role of interleukin 14, 38, 39, and 40 in cancer, and we need more studies to elucidate the exact roles of IL-40 in various kinds of cancerous cells.

### 5.2. IL-40 and Sjogren's Syndrome

According to this, researchers examined the expression of IL-40 at both levels of mRNA and protein in patients with pSS-associated non-lymphoma Hodgkin's (pSS-associated NHL) and primary Sjogren's syndrome (pSS). Interestingly, the expression of IL-40 was noticeably enhanced in two types of patients compared with controls [18]. In support of the role of IL-40 in inflammation, studies showed that in the presence of the anti-inflammatory cytokine like IL-38 in the coculture of human respiratory epithelial cells with macrophages, C17orf99 was downregulated [15].

In another new investigation in 2023, it has been shown that recombinant IL-40 (rIL-40) stimulates the upregulation of proinflammatory cytokines in peripheral pSS T cells. In this experiment, the production of TNF-*α* and IL-17 in PBMCs obtained from pSS patients was assessed after 24 hours incubation with rIL-40. The results indicated that the production of TNF-*α* was significantly increased in both CD_4_^+^ and CD_8_^+^ T cells in pSS patients, but rIL-40 enhanced production of IL-17 only in CD_8_^+^ T cells. These findings indicate that IL-40 may have the potential to activate T cells and promote the production of proinflammatory cytokines in pSS patients. They also showed that rIL-40 induces the production of IFN-*γ* in CD19+ B cells from pSS patients, but no significant role in B cell polarization was found [21]. This evidence suggests an important regulatory influence of IL-40 on both innate and adaptive immunity in pSS patients, raising the possibility that IL-40 can be used as a diagnostic biomarker.

### 5.3. IL-40 and Rheumatoid Arthritis

**Figure 2:**
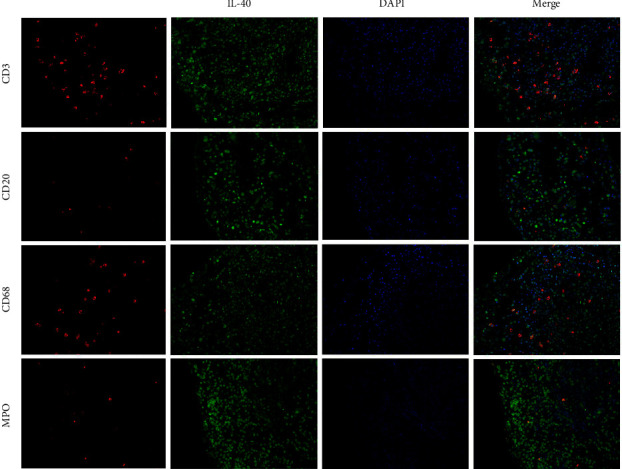
Adapted from [22]. IL-40 is expressed by immune cells in the synovial membrane of patients with rheumatoid arthritis (RA). In addition to the synovial lining, IL-40 positivity (green) was observed in infiltrating cells of RA synovial tissue, demonstrated by specific marker staining (red) for T lymphocytes (CD3), B-lymphocytes (CD20), macrophages (CD68), and neutrophil myeloperoxidase (MPO). Nuclei were stained by DAPI (blue). Representative images of immunofluorescence staining are shown at ×200 magnification (RA, *n* = 4). DAPI, 4′, 6-diamidino-2-phenylindole.

**Figure 3:**
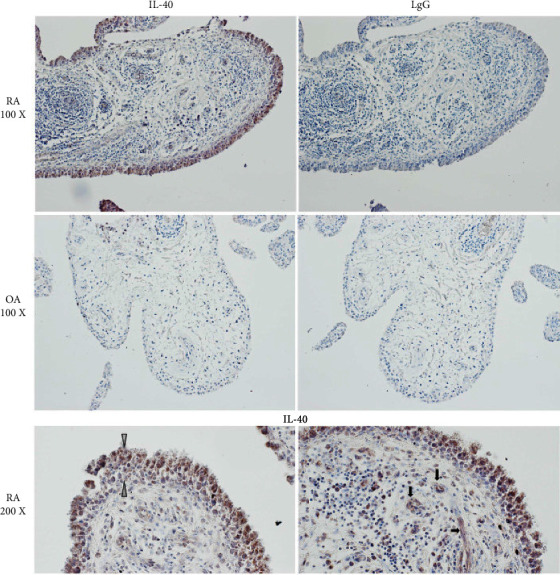
Adapted from [22]. IL-40 in the synovial membrane of patients with rheumatoid arthritis (RA) and osteoarthritis (OA). Intensive IL-40 positivity was observed in RA, especially in the hyperplastic-lining layer and within the inflammatory infiltrates of the RA synovium. Moderate expression of IL-40 was detected in the endothelial cells. IL-40 expression was quite sparse with only few IL-40 positive immune cells within the OA synovial tissue. Rabbit IgG was used as an isotype control. The white arrows point to the hyperplastic-lining layer, and the black arrows point to capillaries. Representative images of immunohistochemistry staining are shown at ×100 magnification, detailed view at ×200 magnification (RA, *n* = 5; OA, *n* = 4).

As B cells play a significant role in most autoimmune and inflammatory diseases like rheumatoid arthritis (RA), several studies have concentrated on the discovery of the role of IL-40 in the pathogenesis of RA. In 2021, one study illustrated that immune cells in the synovium of RA patients expressed a high level of IL-40 (Figure 2) [22]. They also showed that following the B cell-depleting therapy, the production of IL-40 decreased. In other part of this study, they examined the IL-40 expression in the synovial tissue from RA and osteoarthritis (OA) patients. The results showed the expression of IL-40 with immune cells infiltration in synovium tissues of both RA and OA patients, but the expression of IL-40 and immune cells infiltration was significantly higher in RA synovium tissue in compare with OA (Figure 3).

In addition, in 2022, another study showed the high level of IL-40 in blood samples of RA patients, approving its possible diagnostic function in RA [23]. Recently scientists have shown that there is a correlation between rs2004339 A/A, of the gene encoding interleukin-40, C17orf99, and RA risk in Iraqi women. Moreover, it has been shown that IL-40 serum levels in patients were positively influenced by the variant AA genotype [24].

### 5.4. IL-40 and Ankylosing Spondylitis

Ankylosing spondylitis (AS) is another common autoimmunity disorder, which affects the joints. AS is a type of arthritis that causes inflammation in joints and some parts of the spine and affects up to 1 in 100 people. Since it is considered that chronic inflammation has a great role in pathogenesis of AS, evaluating the serum level of IL-36*α*, IL-37, IL-38, IL-39, and IL-40 illustrated that regardless of age, age at disease onset, disease duration, disease activity, or HLA-B27 status, the serum levels of all mentioned cytokines were upregulated in AS patients [25]. In this study, serum levels of IL-40 were measured using an enzyme-linked immunosorbent assay kit in 110 men with AS and 103 healthy control men. The results showed that there was a significant difference in the serum levels of IL-40 between the AS and healthy control groups (*p* < 0.001).

**Figure 4:**
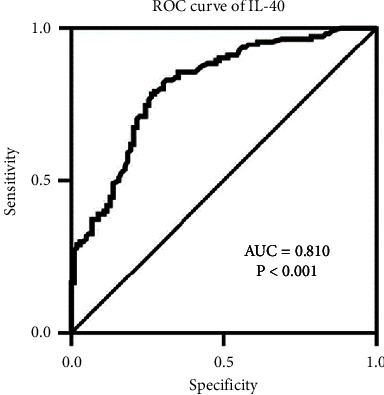
Adapted from [24]. ROC curve analysis of interleukin IL-40 in ankylosing spondylitis patients (area under the curve = 0.810; probability <0.001).

They also investigated the potential of IL-40 as a diagnostic biomarker in AS patients using ROC curve analysis to display the area under the curve (AUC). The AUC refers to the overall performance of a diagnostic marker to distinguish between cases with and without the disease or a condition-based test. An AUC of 0.50–0.59 suggests no discrimination, 0.60–0.69 indicates poor discrimination, 0.70–0.79 is considered acceptable, 0.80–0.89 is considered excellent, and ≥0.9 is considered outstanding. The AUC results illustrated an outstanding relationship between serum levels of IL-40 and probability of AS (Figure 4).

### 5.5. IL-40 and Systemic Lupus Erythematosus

Another immune disease which scientists examined the role of IL-40 in pathogenesis was systemic lupus erythematosus. Systemic lupus erythematosus (SLE) is one of the most common autoimmune diseases and since it is characterized by excessive inflammatory conditions in the body, it is possible that IL-40 plays an important role in pathogenesis or severity of it. Scientists have shown that serum levels of IL- 40 are considerably higher in patients with SLE compared to controls. Moreover, there is a relationship between the serum level of IL-40 and the severity and duration of symptoms in lupus, while the results demonstrated that the mean serum level was highest in the active severe group of patients [26]. This study showed a negative association between levels of IL-40 in serum and C3 and C4 titers and a positive relationship between IL-40 and ESR levels in serum. They also demonstrated an inverse correlation between serum levels of IL-40 in patients who did not take DMARDs (disease-modifying antirheumatic drugs). Therefore, DMARDs may have a protective role by decreasing levels of IL-40 in lupus patients and this could support the potential role of IL-40 in the development of lupus.

### 5.6. IL-40 and Type 2 Diabetes

**Figure 5:**
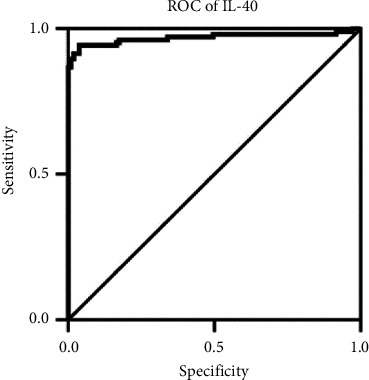
Adapted from [26]. ROC curve analysis of interleukin 40 (IL-40) in type 2 diabetes mellitus (T2DM) patients (area under the curve = 0.969; 95% confidence interval = 0.942–0.997; probability <0.001; cutoff value = 18.7 pg/mL; Youden index = 0.95; sensitivity= 94.3%; specificity = 94.5%).

Complying with this hypothesis about the role of IL-40, an interesting study conducted in 2023 aimed to determine the role of IL-40 as a biomarker of type 2 diabetes, a low-grade inflammatory and metabolic disease. This research by using an enzyme-linked immunosorbent assay revealed that patients had significantly higher levels of IL-40 in serum as compared to controls [27]. This is the only study about the role of IL-40 in type 2 diabetes and showed a 53.36-fold increased risk in patients with T2D in comparison with the control group. Analysis of the ROC curve of IL-40 in type 2 diabetic patients revealed a significant association between elevated IL-40 levels and T2D (Figure 5) (area under the curve = 0.969; probability <0.001; sensitivity = 94.3%; and specificity = 94.5%).

### 5.7. IL-40 and Autoimmune Thyroid Disease

The immune system's malfunction causes autoimmune thyroid disease (AITD), which includes Graves' disease (GD) and autoimmune hypothyroidism (AIH). Recently, the research results indicate that IL-38 and IL-40 serum levels could be promising new diagnostic indicators for patients with GD and AIH. According to the results of this study, levels of IL-40 significantly increased in serum samples of GD and AIH. In addition, it was found that individuals with the GG and CC genotypes of IL-38 and IL-40 might have an increased susceptibility to developing GD and AIH in the Iraqi population [28].

### 5.8. IL-40 and Hepatic Cell Carcinoma

Besides autoimmune disease, IL-40 may also play a crucial role in increasing the inflammation in some cancers. One of the most prevalent forms of primary liver cancer is hepatic cell carcinoma (HCC), and the absence of an effective early diagnosis for HCC is a significant public health concern worldwide. People with chronic liver disease, such as cirrhosis due to hepatitis B, are more likely to develop HCC. Examination of serum levels of IL-40 and pentraxin-3 (PTX3) in patients with HCC showed higher serum levels of PTX3 and IL-40 compared with controls, suggesting that these two factors may be involved in the disease. It is unclear whether IL-40 plays an active role in HCC or not, but several studies have found that the severity of liver inflammation is the only factor that affects the development of HCC. Therefore, a balance between inflammatory and ant-inflammatory cytokines could be considered as an important factor in HCC [29].

## 6. Therapeutic Targets of IL-40

Not only have several scientists focused on the importance of B cells in autoimmunity in recent years but also have many treatments. As a prime example, rituximab has been authorised for the treatment of numerous autoimmune diseases [30]. Lately, another anti-CD20 antibody has been accepted for multiple sclerosis [31]. All of these studies illustrate that B cells play a significant role in autoimmunity or inflammatory diseases, and we can alleviate the progression or severity of these kinds of diseases by eliminating them. On the other hand, as all these remedies impact activated B cells and not plasma cells, the probable effector function of active B cells is cytokine production. From this attitude, the identity of a unique B cell-associated cytokine is a completely fascinating development [32].

To date, there have been no studies on targeting IL-40 for disease treatments or its possible side effects as a drug, and existing researches have primarily focused on identifying the presence of IL-40 in different inflammatory disorders. In patients with early-stage rheumatoid arthritis, only a few studies have indirectly shown that the production of interleukin-40 in patients' serum decreases during 3 months of treatment with conventional drugs [17]. Therefore, in addition to demonstrating the role of this cytokine in various diseases, it may also be useful in the future to conduct studies to determine the role of using this molecule as a therapeutic target.

## 7. Conclusion and Recommendations

Accurate diagnosis in early stages is essential to decrease morbidity or disability in autoimmune and some inflammatory diseases. Unfortunately, due to the absence of a single specific test and similarity of symptoms in autoimmune diseases, physicians sometimes lose the golden time to make a diagnosis. Hence, this issue could be addressed by exploring more specific molecules for diagnosis. All these new studies indicate that there is a significant relationship between IL-40 in serum and the occurrence of RA, SLE, pSS, lymphoma, and other possible diseases, which may not have been discovered yet, and they support the use of IL-40 as a potential biomarker for diagnosis. The following points are recommended, as they are important to acquire more information about IL40:

1. Due to the multiple specific and nonspecific factors involved in autoimmune, cancer, and inflammatory diseases, detailed research is necessary to understand the critical role of IL-40 in disease progression.
2. The role of IL-40 and its correlation with other pro- and anti-inflammatory cytokines should be studied in other diseases as well.
3. Many infectious diseases have inflammatory conditions as well, and IL-40's function in these cases needs to be investigated.
4. The molecule's therapeutic efficacy and potential adverse effects on the body require further investigation. Therefore, a follow-up study is also needed.

In conclusion, a complete understanding of function and role of IL-40 could lead to the identification and treatment of a broad range of inflammation-related illnesses. We are confident that the near-future research will demonstrate the very exciting biology associated with IL-40 in human diseases.

## Figures and Tables

**Table 1:** Summarized characteristic of interleukin 40.

Cytokine	Other names	Gene superfamily	Chromosome location	Receptor	Functions	Main human expression tissues	Target cells	Potential role in diseases
Interleukin-40	C17orf99	None	17q25.3	Unknown	Involved in IgA production, B cell homeostasis and development of humoral immune response	B.M, fetal liver, activated B cells	B cells	B cell lymphoma, lupus, type 2 diabetes, rheumatoid arthritis, Sjogren's syndrome

## Data Availability

No data were used to support the findings of this study.
